# Haemodynamic Changes in the Superior Mesenteric Artery Induced by Acupuncture Stimulation on the Lower Limbs

**DOI:** 10.1155/2012/908546

**Published:** 2012-05-23

**Authors:** Masashi Watanabe, Shin Takayama, Yoshiko Yamamoto, Satoru Nagase, Takashi Seki, Nobuo Yaegashi

**Affiliations:** ^1^Department of Traditional Asian Medicine, Graduate School of Medicine, Tohoku University, Miyagi 980-8575, Japan; ^2^Department of Obstetrics and Gynecology, Graduate School of Medicine, Tohoku University, Miyagi 980-8575, Japan

## Abstract

Acupuncture is commonly performed on acupoints. A comparison of quantitative physiological alterations induced by stimulation on different acupoints has never been performed in the superior mesenteric artery (SMA) in humans. Therefore, we investigated changes in blood flow volume (BFV) in the SMA as an indicator of physiological effects induced by stimulation on 3 points. Thirty healthy participants aged 29 ± 10 years (mean ± SD) were enrolled. All participants underwent stimulations on 3 points located in the lower legs: ST36, LR3, and a non-acupoint. Control pertains to a condition with no-stimulation. Stimulation was performed bilaterally with manual rotation of the needles. BFV was measured by ultrasonography before insertion and 10, 20, 30, and 60 minutes after stimulation. Following acupuncture on ST36, BFV increased significantly 20 and 30 minutes after stimulation, compared to BFV before insertion (*P* < 0.05). Following stimulation on LR3 and the non-acupoint, no significant differences in BFV could be found. Relative to the no-stimulation group, stimulation on LR3, and the non-acupoint, stimulation on ST36 elicited a significant increase in BFV (*P* < 0.05). The results suggest that stimulation on the different points causes distinct physiological effects in BFV in the SMA.

## 1. Introduction

Acupuncture is one of the important parts in traditional Chinese medicine (TCM) [1]. Acupuncture treatment is accomplished through its connection network, the meridian system. This is a vast web of qi and blood activity that pertains to Zang-Fu organs internally and connects to extremities and joints externally [2]. The theory of the meridians is a key component of the theoretical system of TCM [3]. The meridians are pathways along which qi and blood circulate throughout the body, reach the internal organs and limbs, and regulate the thoroughfare vessels [4]. Acupuncture points (acupoints) are gateways located on meridians that can restore the flow of qi [5]. Treatment of specific acupuncture points is based on the diagnosis [6]. The acupuncture theory stands on the meridian system that connects acupoints to the organs. However, it has been difficult to verify the meridian or acupoint structure and the connection between organs and meridians.

 According to the TCM theory, the main physiological functions of the stomach include receiving, digesting, transforming water and food, and taking charge of sending down the transformed food [3]. The Zusanli (ST36) acupoint is located on the stomach meridian. This acupoint is known to be effective in the treatment of digestive system diseases, improving digestive function, and decreasing abdominal pain [1, 3]. One of the functions of the liver is to regulate the free movement of qi. Stagnation of liver qi may impede blood circulation [3] and cause frequent limb chills. The Taichong (LR3) acupoint is the source point of the liver meridian, and stimulation on this point can regulate liver function [1, 3]. However, evidence supporting the functions explained previously remains intangible.

Blood flow volume (BFV) is known to be an important index to demonstrate the condition of organs and tissues. Thus, we employed BFV as a quantitative indicator of the effects of traditional interventions on the human body. There have been some reports about the haemodynamic responses to acupuncture stimulation on several acupoints [7–11]. We reported preliminary data on the effects of acupuncture on BFV in the radial artery [12]. The relationship between peripheral arterial blood flow and cardiac index (CI) has also been studied in human participants with regard to acupuncture [13].

 The superior mesenteric artery (SMA) supplies blood to the entire small intestine, except for the superior duodenum. In addition, it supplies blood to the caecum, the ascending colon, and most of the transverse colon [14]. SMA blood flow pattern and velocity show large variation due to the metabolic activity of the bowel [15, 16]. The BFV of this artery also changes in several diseases [16–21]. It is thought that BFV in the SMA is an important index to evaluate the state of digestive organs. We have previously published a study on the effects of abdominal thermal stimulation and herbal medicine on the BFV in the SMA as measured by ultrasonography [22, 23]. However, to our knowledge, there is no report on the changes in BFV in the SMA induced by acupuncture treatment. Therefore, the aim of this study was to clarify the effects of acupuncture stimulation of 2 different acupoints (ST36 and LR3) and a non-acupoint on BFV in the SMA and CI of healthy participants.

## 2. Methods

### 2.1. Participants

 Thirty healthy adult volunteers (15 men and 15 women) aged  29 ± 10  years (mean ± SD; range, 20–51 years old) were enrolled in this study. All the participants were in good health and had no cardiovascular or abdominal disease. None of the participants took any medicine during the 1-month period before the experiment. All the participants were examined in the morning after fasting overnight and abstaining from alcohol and/or caffeine for at least 10 hours. The study protocol was approved by the Ethics Committee of Tohoku University Graduate School of Medicine. Written informed consent to participation was given by all the participants prior to the beginning of the experiment.

### 2.2. Setting

**Figure 1:**
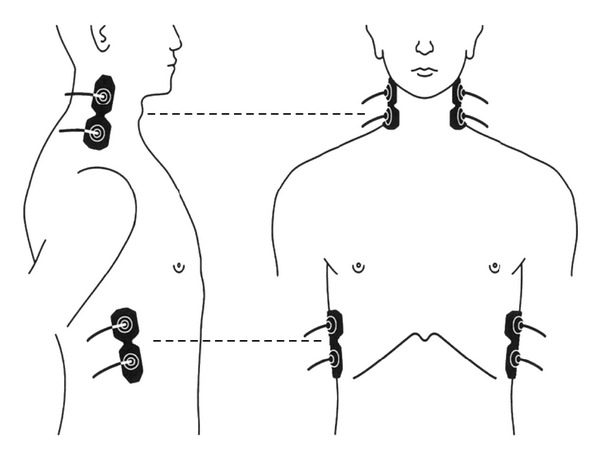
Impedance cardiography (ICG) sensors at the base of the neck and at the level of the xiphoid process in the midaxillary line.

**Figure 2:**
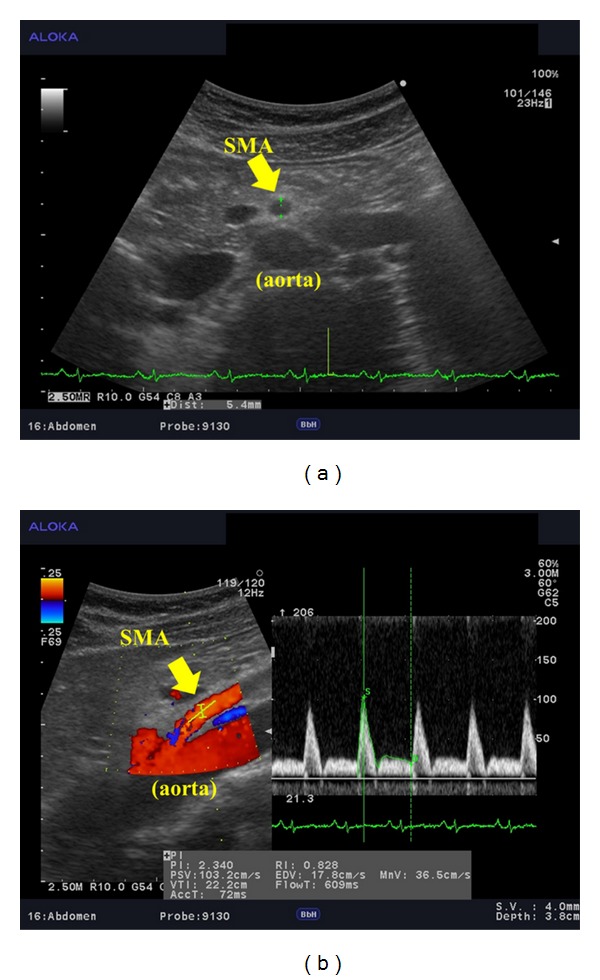
Haemodynamic data obtained by ultrasonography. (a) Measurement of the diameter of the superior mesenteric artery (SMA). (b) Measurement of the blood flow velocity in the SMA.

 The participants rested in the supine position in a quiet, air-conditioned room (temperature, 25-26°C). Three monitoring electrocardiographic electrodes were attached to the anterior part of the chest of each participant. Four electrodes for impedance cardiography (ICG) (Bioz ICG Module, Dash 3000^®^, GE Healthcare, USA) were placed at the base of the neck and at the level of the xiphoid process in the midaxillary line (Figure 1). ICG utilises 4 dual sensors on the neck and chest to apply low amplitude, high frequency, and alternating electrical current to the subject's thorax. Pulsatile changes of BFV and velocity are measured as changes in impedance. It is then synchronised with the electrocardiogram to automatically calculate haemodynamic parameters such as stroke volume and CI [24]. ICG is a noninvasive monitoring method that allows measurement of the CI based on the changes in thoracic resistance that result from variations in intrathoracic BFV [25, 26]. CI is calculated based on the stroke volume, heart rate, and body surface area by ICG [27]. The systemic vascular resistance index was calculated using the CI and blood pressure. Blood pressure was measured with an oscillometer (BP-608 Evolution II^®^, Colin Healthcare Co. Ltd., Kyoto, Japan), on the left upper arm. SMA haemodynamics was measured with an ultrasonography (Prosound *α*10^®^, Hitachi Aloka Medical Ltd., Tokyo, Japan). Combination of the ultrasonography with pulsed Doppler allows the noninvasive investigation of the blood flow in small vessels such as the coronary, splenic, and adrenal arteries, and the SMA [28]. The present ultrasonography had a 5 MHz convex transducer. The vessel diameter (VD) and the distance from the anterior to the posterior intima were used to calculate the cross-sectional area of the SMA (Figure 2). The SMA is the second major branch of the abdominal aorta. It originates just below the celiac trunk, at the ventral wall of the aorta [29]. The SMA measurements were acquired within 2-3 cm from the origin of the artery (Figure 2) [29, 30]. Pulsed Doppler signals were obtained at the same site. For accurate measurement, a Doppler angle of ≤60° was employed [31, 32]. Each Doppler waveform was drawn automatically and was calculated using the software in the ultrasonography. The following haemodynamic parameters were determined: (1) VD (mm), (2) mean flow velocity (MV, cm/s), and (3) blood flow volume (BFV = *π*(VD/2)^2^  × MV, mL/min) [29, 30]. Each parameter was recorded 3 times during 3 different cardiac cycles, and the average for each subject was calculated to minimize errors [30].

### 2.3. Study Protocol

**Figure 3:**
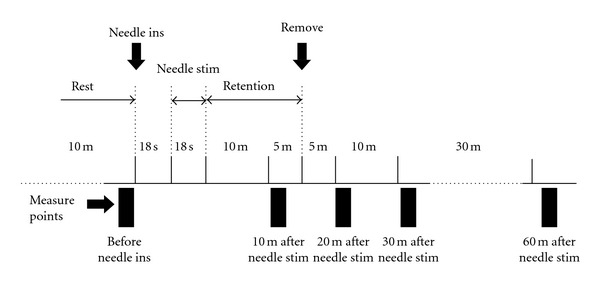
Outline of the study. Needle insertion (ins) and stimulation (stim) were performed bilaterally on ST36, LR3, and the non-acupoint. After the needles were inserted, needle stimulation was applied for 18 s with manual rotation. The needles were retained for 15 minutes after needle stimulation and then removed. Haemodynamic parameters were measured before needle insertion and 10, 20, 30, and 60 minutes after needle stimulation. m: minutes, s: seconds.

**Figure 4:**
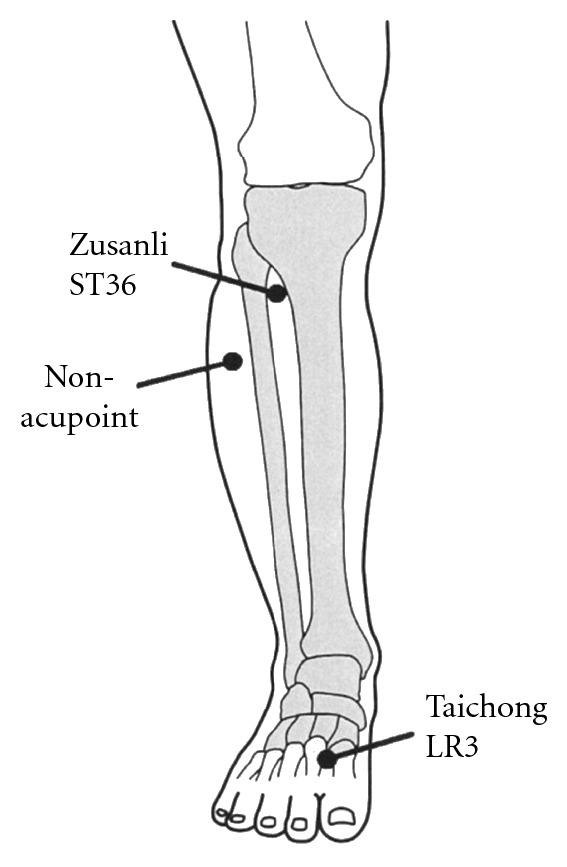
Positions of the needle stimulation point. ST36, LR3, and non-acupoint.

 A randomised single-blind crossover trial was performed. The time course of the study is shown in Figure 3. All participants experienced 4 experimental conditions: (1) needle stimulation on ST36 (Zusanli, located on the lower leg, 3 units below the lateral “eye” of the knee, approximately 1 finger width lateral to the tibia [33]) (Figure 4), (2) needle stimulation on LR3 (Taichong, located on the foot, 1.5–2 units above the web between the first and second toes [33]) (Figure 4), (3) needle stimulation on a non-acupoint (located on the lower leg, 3 units lateral to and below ST36, mid-point of the stomach and gallbladder meridian) (Figure 4), and (4) no-stimulation. The participants had no knowledge about acupuncture or acupoints. All participants underwent each examination in random order. To avoid possible carryover effects, experiments were conducted with an interval of at least 7 days. After positioning of the ultrasonography, the participants rested in the supine position for 10 minutes. We then measured the SMA haemodynamics, blood pressure, heart rate, and CI at rest (before needle insertion) and at 10, 20, 30, and 60 minutes after needle stimulation. Needle stimulation was performed by a licensed acupuncturist. Disposable fine stainless-steel needles (0.16 mm in diameter and 40 mm in length; Serin Co. Ltd., Shizuoka, Japan) were inserted bilaterally on ST36, LR3, and the non-acupoint, and maintained at a depth of 10 mm during the test. After the needles were inserted, stimulation (rotating the needles manually within an angle of 90°) was performed for 18 seconds. The needles were retained for 15 minutes after needle stimulation and then removed.

### 2.4. Statistical Analysis

 Statistical analysis was performed with PASW software (version 17.0; SPSS Japan Inc., Tokyo, Japan). Comparison of acupuncture on ST36, LR3, the non-acupoint, and no-stimulation was performed by two-way analysis of variance (ANOVA). Repeated measures ANOVA, with a post hoc Dunnett's test, was used for statistical comparison between the before needle insertion and after needle stimulation. *P* < 0.05 was considered to be significant. 

## 3. Results

### 3.1. Summary Data

Table 1 summarises the haemodynamic measurements performed before needle insertion and after needle stimulation on ST36, LR3, the non-acupoint, and no-stimulation. This study was a crossover trial. The basal value of BFV in SMA exhibited no significant difference among the 4 groups (*P* = 0.644; one-way ANOVA by PASW verion 17). No significant differences in any of the parameters measured before needle insertion (resting condition) could be found for all tests performed. These results suggest that 7 days after application, the stimulations had no carryover effect.

### 3.2. Blood Pressure, Heart Rate, and Systemic Vascular Resistance Index

Stimulation on ST36, LR3, the non-acupoint, and no-stimulation induced no significant alterations in the blood pressure, heart rate, and systemic vascular resistance index percent before needle insertion or after needle stimulation.

### 3.3. Cardiac Index

**Figure 5:**
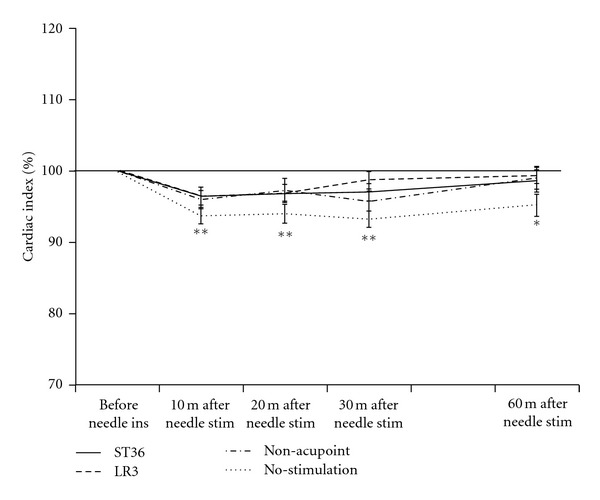
Percentage change in cardiac index following needle insertion (ins) and stimulation (stim) on ST36, LR3, the non-acupoint, and no-stimulation. Values represent the mean and corresponding standard error (SEM). ^∗, ∗∗^Indicate statistical significance (*P* < 0.05, *P* < 0.01, resp.) relative to the resting condition in the no-stimulation setup.

Figure 5 shows the percentage change in CI in each test performed. There were no significant differences among the 4 groups. Stimulation on ST36, LR3, and the non-acupoint induced no significant differences before and after needle stimulation. In the no-stimulation setup, there was a significant decrease in CI before insertion and at 10, 20, 30, and 60 minutes after needle stimulation (*P* < 0.01, *P* < 0.01, *P* < 0.01, and *P* < 0.05, resp.).

### 3.4. SMA Blood Flow Volume

**Figure 6:**
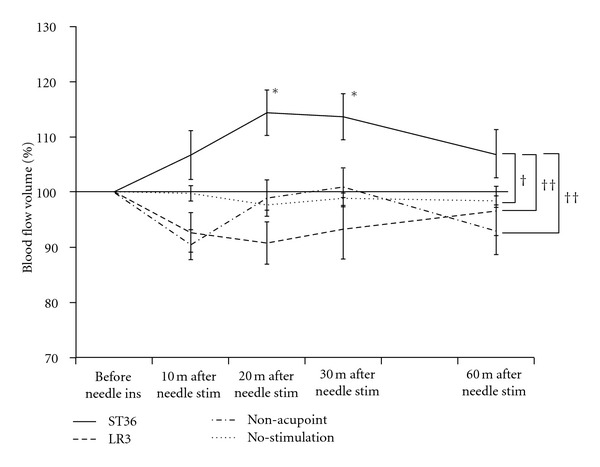
Percentage change in blood flow volume (BFV) in the superior mesenteric artery before needle insertion (ins) and after needle stimulation (stim) on ST36, LR3, the non-acupoint, and no-stimulation. Values represent the mean and corresponding standard error (SEM). *Indicates statistical significance (*P* < 0.05) relative to BFV before needle insertion in ST36. ^†^Indicates statistical significance (*P* < 0.05) between ST36 and no-stimulation. ^††^Indicates statistical significance (*P* < 0.01) between ST36 and LR3/non-acupoint.

Figure 6 shows the percentage change in BFV in the SMA determined in each of the tests performed. In the percentage change in BFV in the SMA, there was a significant difference between the ST36 group and the LR3 group (*P* < 0.01), the ST36 group and the non-acupoint group (*P* < 0.01), and the ST36 group and the no-stimulation group (*P* < 0.05). However, no significant difference in BFV was detected among stimulations on LR3, the non-acupoint, and no-stimulation. Acupuncture on ST36 elicited a significant increase in BFV in the SMA between 20 and 30 minutes after needle stimulation, compared to BFV before needle insertion (*P* < 0.05).

## 4. Discussion

To our knowledge, this is the first report about changes in BFV in the SMA induced by needle stimulation, as assessed using an ultrasonography. The present study revealed that BFV in the SMA significantly increased after needle stimulation on ST36. In contrast, no significant changes were recorded after needle stimulation on LR3, the non-acupoint, and no-stimulation. This result suggests that the physiological effects on the SMA BFV triggered by needle stimulation vary according to the acupoint that is stimulated.

**Figure 7:**
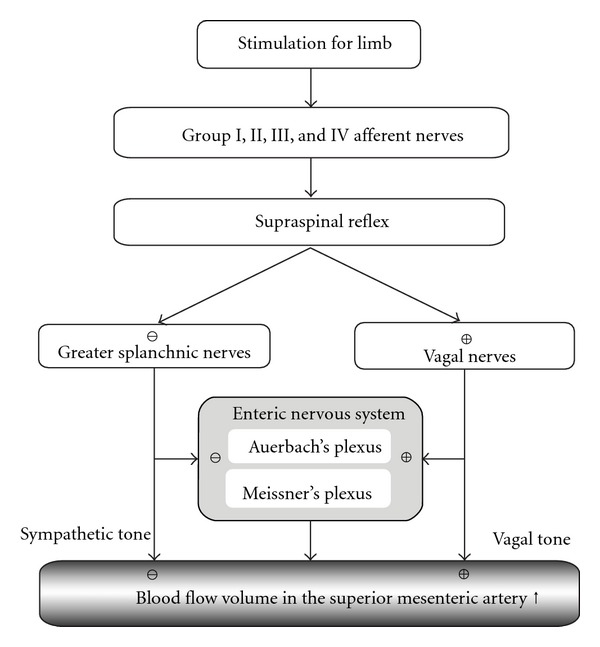
Schematic representation of the hypothetical mechanisms controlling changes in blood flow volume in the superior mesenteric artery. The supraspinal reflex is elicited by stimulation of the limbs, which influence the excitation of vagal nerves or inhibition of sympathetic nerves. An excitation or inhibition is differentiated by the quality of the stimulation. The blood flow volume is influenced by the sympathetic or vagal tone. ⊕: excitation; ⊖: inhibition.

 It is known that BFV in the SMA is regulated by the enteric nervous system and is influenced by the excitation or inhibition of the sympathetic and vagal nerves of the autonomic nervous system [14]. Most, but not all, organs innervated by the autonomic nervous system receive both sympathetic and parasympathetic fibres. The enteric nervous system is, however, a network of neurons intrinsic to the wall of the gastrointestinal tract [14]. It is composed mainly of 2 plexuses: (1) an outer plexus lying between the longitudinal and circular muscle layers, called the myenteric plexus or Auerbach's plexus; and (2) an inner plexus, called the submucosal plexus or Meissner's plexus, which lies in the submucosa [34]. Experimental studies have shown that electroacupuncture inhibition of cardiovascular sympathetic neurons activated through visceral reflex stimulation is performed through the activation of cells in the arcuate nucleus of the hypothalamus, vlPAG in the midbrain, and NRP in the medulla, which in turn inhibit the activity of premotor sympathetic neurons in the rVLM [35]. Acupuncture-like stimulation to the abdomen or a hind limb produces excitatory or inhibitory effects on the adrenal medulla through the sympathetic nerve via a supraspinal reflex pathway. Spinal reflex pathways are tonically inhibited through an inhibitory descending pathway from the brain [36]. We have previously shown that the activity of the gastrointestinal tract can be estimated by measuring BFV changes in the SMA [22, 23]. An increase in the parasympathetic activity or parasympathetic excitation may contribute to an increased BFV in the SMA. A hypothetical mechanism of BFV regulation in the SMA by acupuncture stimulation is presented in Figure 7. Acupuncture on the limbs was also demonstrated to elicit systemic visceral responses via the supraspinal reflexes [36–38]. According to these reports, BFV in the SMA increased significantly after stimulation of the lower limbs [36–38]. We speculate that this increase is caused by excitation of the parasympathetic system and inhibition of the sympathetic system via supraspinal reflexes. However, in this experiment, the BFV in the SMA increased only after stimulation on ST36. Needle stimulation on LR3 and the non-acupoint effected no significant changes in BFV in the SMA. This suggests that stimulation on LR3 and the non-acupoint has no significant impact on the autonomic nervous system, which regulates BFV in the SMA. We thought, therefore, that the various reactions mediated by the autonomic nervous system depend on characteristics of the stimulations, such as the site of the stimulation, intensity of the stimulation, and duration of the stimulation. There is a possibility that the increase in BFV by ST36 acupuncture stimulation is caused by the secondary effect of an increase in gastrointestinal tract motility. It has been demonstrated that the stimulatory effects of acupuncture at ST36 on gastric motility are mediated via vagal efferent in rats [39]. It is conceivable that the stimulatory effects of acupuncture may be mediated via vagal pathway in humans [40]. Moreover, BFV in the gastrointestinal organs is also controlled by the autonomic nervous system. It appears that the simultaneous increases in motility and BFV in these organs are caused by vagal tone. 

 Stimulation points of this study (i.e., ST36, LR3, and the non-acupoint) are all located on the same limb dermatomes [14]. Nevertheless, the reactions induced by needle stimulation on these points were different. The dermatomes of the lower limb arise from spinal nerves T12 to S3 [14]. Considerable overlap exists between adjacent dermatomes innervated by nerves derived from consecutive spinal cord segments [14]. Mori et al. reported that acupuncture on the hind paw and hind leg produced different responses in rats and speculated that the afferents innervating the skin and muscles might have different reflex influences on some visceral organ functions with sympathoadrenal medullary function [41]. Acupuncture stimulation of the limbs may cause systemic and specific reactions in an organ system. The 3 points used in this study (ST36, LR3, and non-acupoint) are located along the boundary of the same dermatomes and their innervation is complex. ST36 is innervated by the lateral cutaneous nerve of the calf at L5, S1, and S2 [14]. LR3 is innervated by the deep fibular nerve and dorsal digital nerve at L5 [14]. The non-acupoint is innervated by the superficial fibular nerve at L4, L5, and S1 [14]. The 3 points exist on different muscles. ST36 is located on the tibialis anterior muscle, which is innervated by the deep fibular nerve at L4 and L5 [14]. LR3 is located on the extensor digitorum brevis muscle, which is innervated by the lateral terminal branch of the deep fibular nerve at L5 and S1 [14]. The non-acupoint is located on the extensor digitorum longus muscle, which is innervated by the deep fibular nerve at L5 and S1 [14]. ST36 and LR3 are located near the muscle tendons, whereas the non-acupoint is located in middle of the muscle. ST36 and the non-acupoint are located in the lower limb, whereas LR3 is located in the foot. The muscle of LR3 is thinner than that of ST36 or the non-acupoint. Therefore, we believe that each point has a different sensitivity to stimulation. It is also of note that differences in signal transmission through afferent and efferent fibres depend, among others factors, on anatomical structures such as the dermatomes and myotomes and on the innervation and thickness of the muscle and skin on the stimulation points. Li and Longhurst [35] reported that other acupoints (e.g., PC5-6) influencing haemodynamic responses may be equally or more effective than ST36. Point specificity has been indicated in other studies on cardiovascular reflex responses. In addition, other studies, based on evaluations by functional MRI, showed that stimulation of limb acupoints located on the same spinal segments induced distinct, though overlapping, cerebral response patterns, indicating the existence of acupoint specificity [42–44]. The present study further supports this, suggesting that responses triggered by acupuncture vary according to the acupoint stimulated, as found for the effects of stimulation on ST36 and LR3 in BFV in the SMA. In this experiment, there were no significant differences in blood pressure and heart rate on ST36, LR3, or the non-acupoint before and after needle stimulation. Li et al. have reported the hypotensive effects of electroacupuncture on ST36 and ST37 and the nonhypotensive effects by electroacupuncture on GB37 and GB39 in subjects with mild-to-moderate hypertension [35, 45]. However, Longhurst reported that acupuncture in normotensive human subjects does not alter blood pressure [46]. The current study was conducted in healthy subjects; therefore, our results regarding cardiovascular reactions support the latter study. The effects of acupuncture in the current study lasted 15 minutes. Other studies have shown that the prolonged acupuncture actions can last up to one and half hour [35]. These studies suggest that reciprocal excitatory projections between the arcuate nucleus and the vlPAG may form a reinforcing circuit that can be activated for prolonged periods by electroacupuncture, with effects lasting for as long as 30–60 minutes [35]. We believe that the cardiovascular reaction is dependent on acupoint location, quality of stimulation, quantity of stimulation, and the sensitivity of the subjects.

The present result showed a significant decrease of CI in the no-stimulation setup at each measuring point of the rest. We thought that significant decrease in CI in this group was elicited by the long-term rest without stimulation. On the other hand, no significant differences in the CI were measured before and after needle stimulation on ST36, LR3, and the non-acupoint. Electroacupuncture applied for 30 minutes stimulating the somatic nerves and supraspinal regions modifies cardiovascular responses but does not alter coronary blood flow [47]. The gentle painless stimulation performed in this study may have elicited to maintain the CI.

 Our trial was an observational study in humans. It is difficult to measure sympathetic and parasympathetic activities invasively in human subjects. Therefore, we observed the reaction in BFV at 2 acupoints and a non-acupoint and speculated on the mechanism using previous experimental studies. Spectral analysis of RR variability is useful to evaluate the autonomic nervous balance noninvasively. However, we did not perform continuous electrocardiography; thus, spectral analysis of RR variability could not be evaluated. We would like to conduct further research using this test in the next study.

## 5. Conclusions

 In the present study, we have shown an increase in BFV in the SMA after needle stimulation on ST36 without change in CI. Needle stimulation either on LR3 or on the non-acupoint had no influence on BFV in the SMA. Physiological effects may be dependent on acupoint location, quality of stimulation, quantity of stimulation, and the sensitivity of the subjects.

## Figures and Tables

**Table 1:** Summary of the haemodynamic parameters measured. Values represent the mean and corresponding standard deviation (SD). ins: insertion; stim: stimulation.

Parameter/test condition	Before needle ins	10 min after needle stim	20 min after needle stim	30 min after needle stim	60 min after needle stim
Systolic blood pressure (mmHg)					
ST36	111.6 ± 9.8	109.1 ± 8.3	108.8 ± 9.5	109.0 ± 8.4	112.6 ± 8.8
LR3	110.4 ± 9.5	109.8 ± 11.0	109.0 ± 9.2	109.8 ± 9.3	111.2 ± 11.4
Non-acupoint	110.0 ± 7.6	108.0 ± 9.2	107.7 ± 9.4	108.5 ± 8.5	109.2 ± 6.9
No-stimulation	110.2 ± 10.2	109.7 ± 10.4	107.4 ± 8.3	108.7 ± 10.4	108.4 ± 9.4
Diastolic blood pressure (mmHg)					
ST36	66.0 ± 8.1	65.1 ± 7.1	66.0 ± 8.6	64.4 ± 6.8	67.6 ± 7.0
LR3	66.7 ± 7.7	65.4 ± 7.9	65.5 ± 7.4	65.3 ± 6.6	67.7 ± 7.8
Non-acupoint	64.6 ± 6.6	64.1 ± 6.6	63.2 ± 6.6	63.1 ± 7.6	64.9 ± 7.2
No-stimulation	65.1 ± 7.0	62.7 ± 8.7	63.7 ± 7.2	64.0 ± 6.4	64.6 ± 8.3
Heart rate (beats/min)					
ST36	58.4 ± 8.6	57.0 ± 7.5	57.4 ± 8.2	57.8 ± 8.1	57.2 ± 7.0
LR3	58.2 ± 8.6	57.5 ± 8.2	57.7 ± 7.9	57.4 ± 7.4	58.2 ± 8.0
Non-acupoint	57.7 ± 7.3	55.8 ± 6.6	56.6 ± 6.4	56.7 ± 6.5	57.8 ± 6.4
No-stimulation	57.9 ± 10.2	56.4 ± 8.1	56.9 ± 7.9	56.8 ± 7.8	57.3 ± 7.5
Cardiac index (L/min/m^2^)					
ST36	2.7 ± 0.3	2.6 ± 0.3	2.6 ± 0.2	2.6 ± 0.3	2.6 ± 0.3
LR3	2.7 ± 0.3	2.6 ± 0.2	2.6 ± 0.3	2.6 ± 0.2	2.6 ± 0.2
Non-acupoint	2.7 ± 0.4	2.6 ± 0.2	2.6 ± 0.3	2.6 ± 0.3	2.7 ± 0.3
No-stimulation	2.7 ± 0.4	2.5 ± 0.3	2.6 ± 0.3	2.5 ± 0.3	2.6 ± 0.3
Systemic vascular resistance index (dyne sec/cm^5^ m^2^)					
ST36	2415 ± 333	2467 ± 297	2474 ± 324	2439 ± 309	2509 ± 311
LR3	2471 ± 371	2534 ± 382	2525 ± 449	2471 ± 323	2517 ± 362
Non-acupoint	2401 ± 351	2468 ± 322	2413 ± 308	2454 ± 301	2424 ± 322
No-stimulation	2397 ± 320	2514 ± 323	2495 ± 337	2527 ± 325	2506 ± 367
Blood flow volume in superior mesenteric artery (mL/min)					
ST36	729.2 ± 354.2	777.1 ± 436.3	835.1 ± 437.3	828.4 ± 460.3	772.6 ± 401.4
LR3	866.3 ± 518.6	788.6 ± 486.9	780.3 ± 462.2	813.1 ± 548.2	828.8 ± 488.3
Non-acupoint	826.7 ± 484.2	743.2 ± 418.1	815.3 ± 487.5	814.3 ± 438.7	779.8 ± 525.3
No-stimulation	833.7 ± 434.9	809.8 ± 396.1	784.3 ± 390.1	785.8 ± 399.4	821.1 ± 485.0
